# Second-order nonlinear optical switching with a record-high contrast for a photochromic and thermochromic bistable crystal†
†Electronic supplementary information (ESI) available: Crystallographic data, bond lengths and angles, and characterization of **1**. CCDC 1519422. For ESI and crystallographic data in CIF or other electronic format see DOI: 10.1039/c7sc01228d


**DOI:** 10.1039/c7sc01228d

**Published:** 2017-09-20

**Authors:** Xiu-Shuang Xing, Rong-Jian Sa, Pei-Xin Li, Ning-Ning Zhang, Zhong-Yuan Zhou, Bin-Wen Liu, Jie Liu, Ming-Sheng Wang, Guo-Cong Guo

**Affiliations:** a State Key Laboratory of Structural Chemistry , Fujian Institute of Research on the Structure of Matter , Chinese Academy of Sciences , Fuzhou , Fujian 350002 , P. R. China . Email: mswang@fjirsm.ac.cn ; Email: gcguo@fjirsm.ac.cn; b University of Chinese Academy of Sciences , Beijing 100039 , P. R. China

## Abstract

Nonlinear optical (NLO) switchable materials are important for photonic and optoelectronic technologies. One important issue for NLO photoswitching, the most studied physical switching approach, is how to improve the switching contrast of second harmonic generation (SHG) in crystals, because the known values are generally below 3 times. Thermoswitching, as another approach, has shown impressive high SHG-switching contrasts (4–∞ times), but the fast decay of thermally induced states demands constant heat sources to maintain specific SHG intensities. We have synthesized a photochromic and thermochromic bistable acentric compound, β-[(MQ)ZnCl_3_] (MQ^+^ = *N*-methyl-4,4′-bipyridinium), which represents the first crystalline compound with both photo- and heat-induced SHG-switching behavior and the first example of a thermoswitchable NLO crystal that can maintain its expected second-order NLO intensity without any heat source. The SHG-switching contrast can reach about 8 times after laser irradiation or 2 times after thermal annealing. The former value is the highest recorded for photoswitchable NLO crystals. This work also indicates that higher SHG-switching contrasts may be obtained through increasing electron-transfer efficiency, variation of permanent dipole moment, and self-absorption.

## Abstract 2

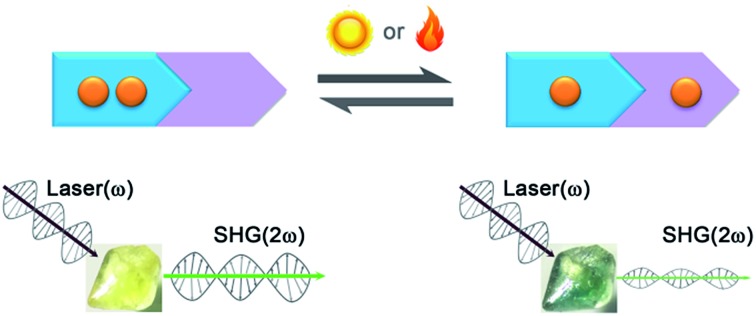
The first bistable NLO crystalline compound with both photo- and heat-induced SHG-switching behavior shows a record-high photoinduced SHG-switching contrast.

## Introduction

NLO effects, such as SHG, SFG, DFG, OPO and multiphoton absorption, have found important applications in signal processing, memory, biological imaging and other appealing fields.^1^ In the past few years, materials displaying switchable NLO properties have drawn much attention.^2^–^10^ On one side, they provide an *in situ*, reversible, and controllable method for regulating NLO properties. This method may be applied in the currently emerging nanolaser field to adjust laser intensity.^11^ On the other side, the switchable NLO properties can behave as readout signals for sensing, data storage, and other applications. For instance, they can be applied in non-destructive data storage of photochromic materials, because light of long wavelengths outside of the absorption band does not induce photoreaction.^12^

**Scheme 1:**
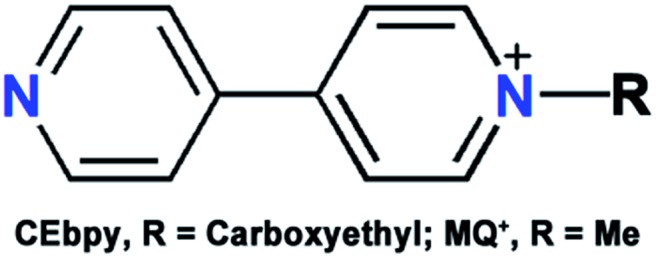
The structures of MQ^+^ and CEbpy.

There have been many approaches to switching the SHG effect. Ion exchange^13^,^14^ and gas adsorption/desorption^15^ methods have been found to be effective for porous materials. Compared with these chemical methods, physical methods such as irradiation and heating are more convenient and applicable to more material systems. As the most studied physical approach, SHG photoswitching has been almost realized in liquid or film photochromic materials with the presence of external optical^16^–^19^/electric^8^,^20^–^23^ fields or special supported media.^24^–^26^ For example, the Guerchais group reported photoinduced SHG-switching properties of some photochromic dithienylethene-based platinum(ii) complexes in solution measured by the EFISH technique under an electric field.^8^ Second-order NLO crystals do not require the above severe conditions to switch the SHG effects owing to their intrinsic polarities. For instance, Sliwa and coworkers found that photochromic anil molecules in the crystalline state exhibited SHG effects and SHG-switching properties without the induction of any external stimuli.^3^ However, in an early research effort, photoswitching of the SHG effects in NLO crystals was fulfilled through large molecular isomerization reactions of organic photochromic components.^3^,^27^–^29^ The large structural change needs sufficient steric space and is usually prohibited in the crystal lattice.^30^ We^31^,^32^ and the Mercier group^33^ previously found through single-crystal X-ray diffraction analysis, that electron-transfer (ET) photochromic processes^34^ are only accompanied by minor structural variation, which is more adaptable to a constrained medium. These processes result in the rearrangement of charges and thus change the permanent dipole moment, which is closely related to the SHG effect of a crystal. On the basis of these aspects, we have recently successfully synthesized the first ET photochromic crystalline compound, [ZnBr_2_(CEbpy)]·3H_2_O (CEbpy = *N*-carboxyethyl-4,4′-bipyridinium, Scheme 1), with photoswitchable second-order NLO properties.^35^ The observed SHG-switching contrast of ∼3.3 times is larger than those of previously reported photoswitchable NLO crystals (∼1.1–2.5 times).^3^,^27^–^29^ Thereafter, the Zang group reported that ET photochromism was also rather effective in switching the SHG efficiency of a crystalline viologen-functionalized chiral Eu-MOF, giving a SHG-switching contrast of ∼2.5 times.^36^ Even so, the relative studies are just starting out, and it is still desirable to achieve NLO crystals with higher SHG-switching contrasts.

**Fig. 1:**
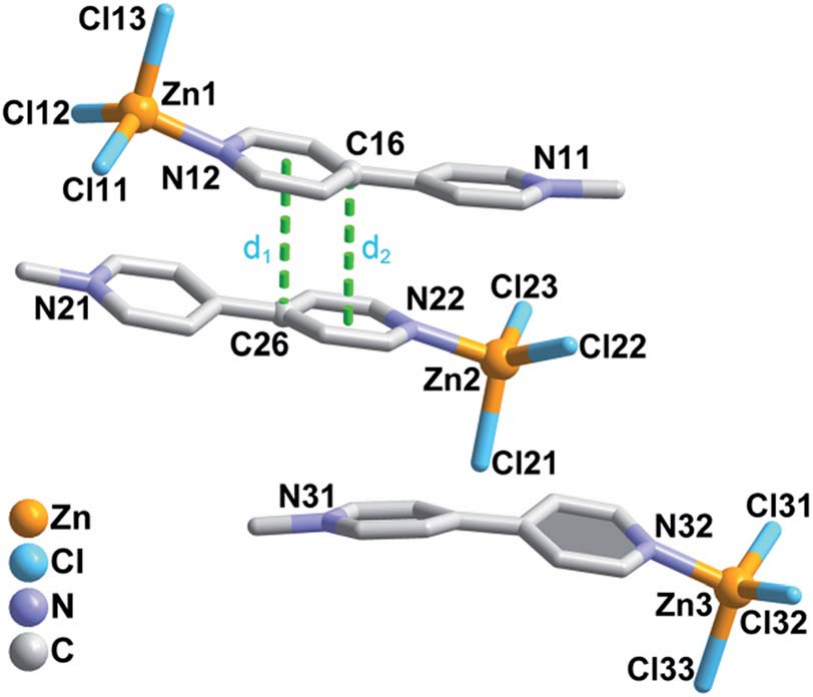
Asymmetric structural unit of **1**. Centroid (pyridyl, N12)-to-C26 and C16-to-centroid (pyridyl, N22) separations: *d*_1_ = 3.630(5) Å; *d*_2_ = 3.574(5) Å. H atoms are omitted for clarity.

Compared with photoswitching, thermoswitching of SHG effects in the crystalline state has fewer reported examples.^4^,^9^,^37^–^43^ Luo’s group^9^,^38^–^43^ found that thermally induced order–disorder phase transitions of DABCO salts (DABCO = 1,4-diazabicyclo[2.2.2]octane), crown ether derivatives and propanediol analogues resulted in SHG-switching contrasts of 12–150 times. Mercier’s group reported that organic disulfide-based metal halides displayed thermoswitching of SHG signals from active to silent due to conglomerate-to-racemate transition.^4^,^37^ Sliwa and coworkers revealed that intramolecular enol–keto tautomerism of thermochromic Schiff bases yielded a SHG-switching contrast of 4 times.^44^ All of these materials exhibit impressive high SHG-switching contrasts, however they require a constant heat source to maintain the expected second-order NLO intensity owing to the fast decay of the thermally induced state. Second-order NLO materials with photochromic and thermochromic bifunctional properties may provide not only both the photoswitching and thermoswitching functions but also stable light/heat-induced states. In this work, using d(+)-Camphoric acid (d-Cam) as an acentric inducer, we synthesized a new photochromic and thermochromic bifunctional compound, β-[(MQ)ZnCl_3_] (**1**; Fig. 1), with an acentric crystal structure that differs from our previously reported centrical one for α-[(MQ)ZnCl_3_]^45^ (α and β denote different phases). The MQ^+^ ligand was selected considering the following three things: (1) *N*-substituted-4,4′-bipyridinium compounds are likely to display both ET photochromic and thermochromic properties;^46^–^52^ (2) its reduced product MQ˙ in the crystalline state could be kept in air for several weeks after removing external stimuli;^45^ and (3) it has a more negative difference in Gibbs free energy and a larger change in permanent dipole moment than those of the above-mentioned CEbpy ligand after receiving one electron (Δ*G*: –5.42 eV for MQ^+^, –2.26 eV for CEbpy; change of permanent dipole moment: –2.62 Debye for MQ^+^, –0.48 Debye for CEbpy; Table S1 in the ESI†), which is indicative of a better ability to stabilize received electrons after photo/heat-induced ET and a higher probability to obtain a large SHG-switching contrast. As we expected, compound **1** displayed unprecedented photo- and thermoswitchable second-order NLO properties, and its switched second-order NLO intensity could be stable in N_2_ for at least one day after removing the external light and heat sources. Note that compound **1** also represents the first thermoswitchable NLO crystal that can maintain the expected second-order NLO intensity without any heat source. The SHG-switching contrast may reach about 8 times after laser irradiation or 2 times after thermally annealing. Notably, the former value is more than twice that of the highest-known record (∼3.3 times) for photoswitchable NLO crystals.^35^ Herein, we would like to present the synthesis, crystal structure, and optical properties of **1**.

## Results and discussion

### Crystal structure

Single-crystal X-ray crystallographic analysis demonstrated that compound **1** crystallizes in the monoclinic acentric space group *Cc*, and that the Flack factor is –0.02(2). As shown in Fig. 1, the asymmetric unit of **1** consists of three neutral isolated [(MQ)ZnCl_3_] molecules. Each zinc atom is four-coordinated by one N atom from the MQ^+^ ligand and three terminal chlorine anions, yielding a distorted tetrahedral configuration. The adjacent [(MQ)ZnCl_3_] molecules containing the Zn1 and Zn2 atoms are assembled in a head-to-tail fashion by offset π–π stacking interactions^53^ between the MQ^+^ ligands to form a π-stacking dimer, with centroid (pyridyl, N12)-to-C26 and C16-to-centroid (pyridyl, N22) distances of ∼3.629 and 3.574 Å, respectively. The [(MQ)ZnCl_3_] molecule with the Zn3 atom (denoted as Zn3-moiety) is nearly parallel to the π-stacking dimer.

### Photochromism and thermochromism

**Fig. 2:**
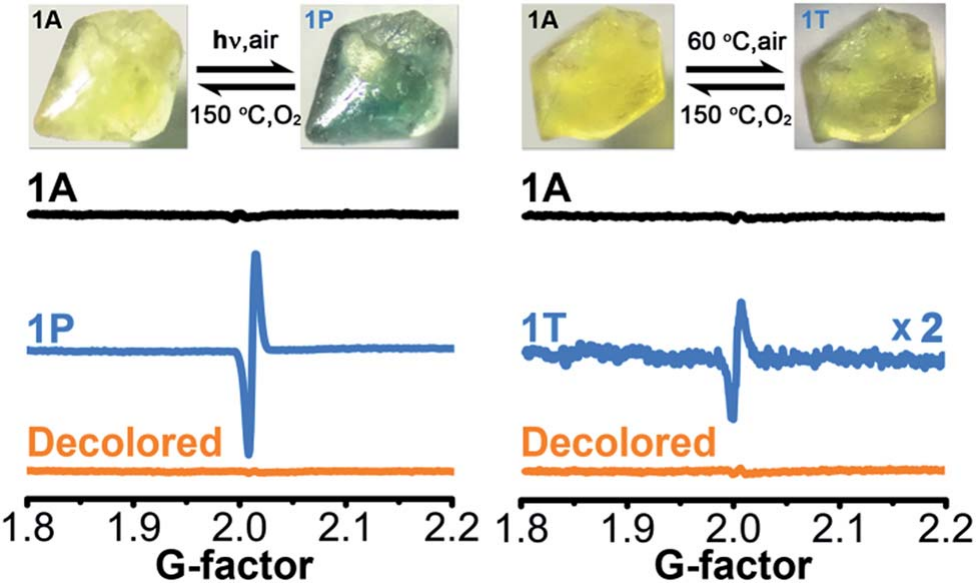
Photochromic and thermochromic phenomena and ESR data of **1** (**1A**, as-synthesized; **1P**, irradiated; **1T**, thermally annealed).

**Fig. 3:**
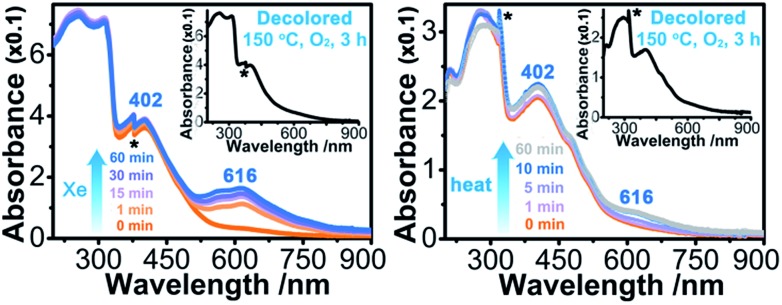
(Left) Irradiated time-dependent UV-vis absorption spectra of **1** measured at room temperature in air using a 300 W Xe lamp (denoted as Xe). Inset: UV-vis spectrum after decoloration. (Right) UV-vis absorption spectra measured at room temperature in air for **1** with different thermal annealing times at 60 °C in air. Inset: UV-vis spectrum after decoloration. Noise is labelled with the * symbol.

Upon continuous irradiation with a PLS-SXE300C 300 W xenon lamp at room temperature in air, the as-synthesized crystalline sample of **1** (denoted as **1A**) showed a color change from pale yellow to green (Fig. 2). The photoresponsive range was around 330–390 nm, and the most efficient wavelength was around 350 nm (Fig. S1 in the ESI†). In the ultraviolet-visible (UV-vis) spectrum (Fig. 3 left), the green photoproduct (denoted as **1P**) displayed a new broad absorption band around *λ* = 616 nm and slightly enhanced absorption at 402 nm, similar to that found for the photochromic compound α-[(MQ)ZnCl_3_].^45^ The intensity of the absorption band was almost unchanged after irradiation for more than 60 min. We used a 532 nm laser to irradiate the photoproduct. No obvious decoloration could be observed, which indicates that the photoinduced decoloration of compound **1** was prohibited. Thermogravimetry (TG) and differential scanning calorimetry (DSC) analysis data (Fig. S2 in the ESI†) showed that compound **1** can be stable up to 330 °C. After annealing at 150 °C in O_2_ for 3 h, the new UV-vis absorption band disappeared (Fig. 3 left, inset) and the initial pale yellow color was regained.

Powder X-ray diffraction (PXRD) data (Fig. S3 in the ESI†), and FT-IR spectra (Fig. S4 in the ESI†) verified that the crystal structure showed no clear variation during the coloration and decoloration processes, and thus the possibility of a photoinduced dissociation or rearrangement reaction can be excluded. An electron spin resonance (ESR) study revealed that **1P** exhibited a strong symmetric signal with a *g* value of 2.0033 and a linewidth of 14 Gauss (Fig. 2). The *g* value is close to that of a free electron found at 2.0023, indicating the formation of radicals.^54^ The absorption band at around 616 nm for **1P** resembles the observed ones for MQ˙ radicals^45^ and other *N*,*N*′-disubstituted or *N*-monosubstituted-4,4′-bipyridinium radicals.^55^,^56^ Therefore, the illumination resulted in the reduction of the MQ^+^ cations and the formation of MQ˙ radicals. Similar to the case found in α-[(MQ)ZnCl_3_],^45^ the Cl atoms should be electron donors.

Sample **1A** also showed a color change upon direct heating. After annealing at 60 °C in air for several minutes, its UV-vis spectrum (Fig. 3 right) underwent a similar change to that upon irradiation (Fig. 3 left), except that the intensity of the 616 nm band increased slowly and quickly reached the maximum value when the heating time was about 10 min. Thus, the color change was not remarkable. Finally, a yellow-green product (denoted as **1T**) was obtained. A characteristic ESR signal for MQ˙ radicals, with a *g* value of 2.0030 and a linewidth of 15 Gauss, was also observed for **1T**, indicating that a Cl → MQ^+^ electron transfer process occurred upon heating as in the case upon irradiation. PXRD (Fig. S5 in the ESI†) and FT-IR data (Fig. S6 in the ESI†) showed that the structure of **1** underwent no obvious changes during the coloration–decoloration processes, and thus the possibility of a heat-induced dissociation or rearrangement reaction can be also excluded. Sample **1T** was completely bleached after annealing at 150 °C in O_2_ for about 3 h (Fig. 3 right, inset). At the same time, a UV-vis absorption spectrum resembling that of **1A** was regained, and the radical signal disappeared in the ESR spectrum (Fig. 2).

### Photo-/thermoswitching of SHG effects

SHG measurements for sieved crystalline samples, using a fundamental laser beam (*λ* = 1064 nm), indicated that the as-synthesized sample **1A** had a macroscopic SHG response approximately 0.1 times that of KH_2_PO_4_ (KDP) and was phase-matchable (Fig. S7 in the ESI†). A CASTEP optical properties calculation at the GGA/RPBE level in a unit cell showed that the phase-matching direction in the crystal is mainly determined by *n*_*z*_–*n*_(*x* or *y*)_ (Fig. S8 in the ESI†), where *n*_*x*_, *n*_*y*_ and *n*_*z*_ are the fractions of the refractive index along the *x*, *y* and *z* direction and the *y* and *z* directions are parallel to the crystallographic *b* and *c* axes, respectively.

**Fig. 4:**
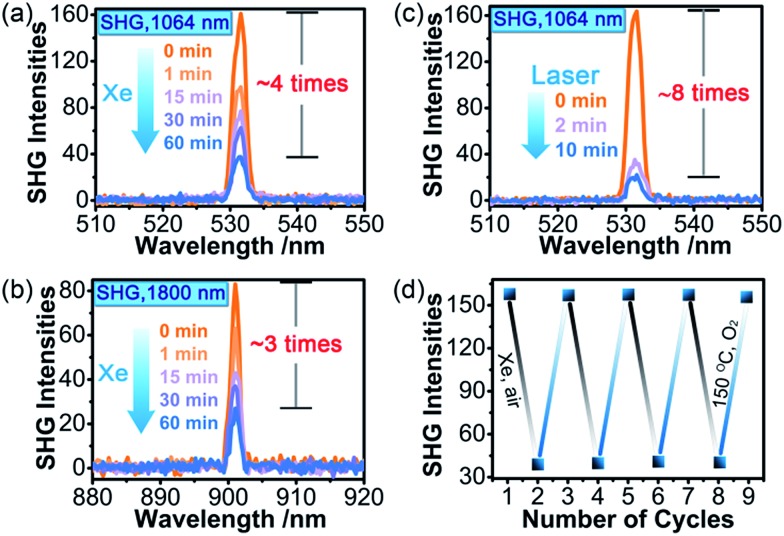
The time-dependent SHG intensity of **1** (particle size: 150–200 μm) upon irradiation at room temperature in air by the 300 W Xe lamp (fundamental laser beam: (a) 1064 nm, 4 mJ; (b) 1800 nm, 3.5 mJ) or the 355 nm DPSS laser ((c); fundamental laser beam: 1064 nm, 4 mJ). SHG switching in four-cycle photochromic processes using the Xe lamp (d). The SHG intensities are the average of one hundred single pulse signals.

**Fig. 5:**
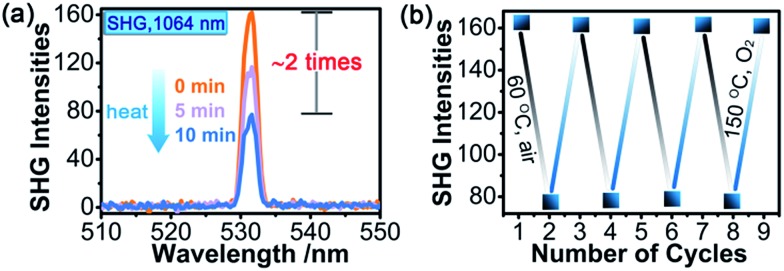
(a) SHG intensity measured at room temperature in air for **1** (particle size: 150–200 μm; fundamental laser beam: 1064 nm, 4 mJ) with different thermal annealing times at 60 °C in air. (b) SHG-switching in four-cycle thermochromic processes. The SHG intensities are the average of one hundred single pulse signals.

The most striking feature of **1** is that its SHG intensity is clearly weakened after photo- and heat-induced coloration. The SHG intensity dropped gradually upon successive irradiation with the Xe lamp, and a maximum SHG-switching contrast of ∼4 times was obtained after 60 min (Fig. 4a). The SHG-switching contrast can be remarkably enhanced when a laser is chosen as the light source. For instance, as shown in Fig. 4c, the SHG-switching contrast reached about 8 times upon irradiation by a Newport Co. Pulseo GKNQL-355-3-30 diode pumped solid state (DPSS) laser (355 nm; 70 kHz; 39 ns pulse width; *ca.* 1.55 mJ cm^–2^; spot size, *ca.* 1.5 cmφ) for 10 min, which is more than twice that of the reported highest value (∼3.3 times) for SHG-switchable crystals.^36^ Moreover, the SHG intensity of **1** became half of the original value after thermal annealing in air at 60 °C for 10 min (Fig. 5a). The photoswitching and thermoswitching of SHG intensity for **1** can be cycled at least four times (Fig. 4d and 5b). UV-vis absorption spectra show that the charge-separation state had no change after keeping the irradiated sample in the dark under N_2_ for 1 d (Fig. S9 in the ESI†), indicating that **1** can maintain the expected second-order NLO intensity without a light/heat source.

The decrease in the SHG intensity after photo- or heat-induced coloration may result from two factors. On one side, as stated in the Introduction, the ET process of **1** causes a remarkable change in permanent dipole moment, which is closely related to its SHG effect. As shown in Table 1, the difference between the calculated permanent dipole moment values before and after ET (spin multiplicity *S*: before, 1; after, 3) is 20.08 (B3LYP) or 20.21 (M06) Debye for the asymmetric structural unit shown in Fig. 1. This value is close to that of the Zn3-moiety and significantly larger than that of the π-stacking dimer, which indicates that the Zn3-moiety contributes mostly to the change in permanent dipole moment for the asymmetric unit. Therefore, the direction of the whole permanent dipole moment for the cell of **1** is basically dependent on the arrangement of the Zn3-moieties. As calculated, the permanent dipole moment for a cell built exclusively by the Zn3-moieties changed from 96.35 (B3LYP)/99.5 (M06) to 82.08 (B3LYP)/84.32 (M06) Debye after ET. A TD-DFT calculation at the B3LYP level showed that the transition dipole moment, oscillator strength, and absorption intensity for the Zn3 moiety became small after electron transfer and coloration when the excitation wavelength was close to 1064 nm (Table S2 and Fig. S10 in the ESI†). Moreover, the static (*λ* = ∞ nm; Table 2 and Table S3 in the ESI†) and dynamic (*λ* = 1800 nm; Tables S4 and S5 in the ESI†)^57^ first hyperpolarizability *β*(–2*ω*; *ω*, *ω*) for the initial state, calculated based on the Zn3-moiety, were larger than those for the colored state. In summary, the permanent dipole moment, oscillator strength, and *β*(–2*ω*; *ω*, *ω*) all become small, in accordance with the observed decrease in SHG intensity. The ESR signal of the photogenerated radical product of **1** is clearly stronger than that of the heat-induced one (Fig. 2). This result indicates that the ET efficiency in the photochromic process is higher than that in the thermochromic process, and the decrease in polarity triggered by light is much larger than that by heat for the crystalline sample of **1**.

**Table 1:** Calculated permanent dipole moment values of structural units in **1** before and after ET. Dipole moment vectors for different components at the closed-shell singlet and open-shell triplet states are labelled in Fig. S11 in the ESI

		Permanent dipole moment/Debye			
		Asymmetric unit	Zn3-moiety	π-Stacking dimer	Cell with only Zn3-moieties^*a*^
Before ET	B3LYP	39.75	40.52	1.65	96.35
	M06	39.91	41.63	1.64	99.50
After ET	B3LYP	19.67	16.03	1.16	82.08
	M06	19.70	15.78	1.15	84.32
Difference	B3LYP	20.08	24.49	0.49	14.27
	M06	20.21	25.85	0.49	15.22

^*a*^A cell with only Zn3 moieties was obtained by removing the Zn1 and Zn2 moieties from the cell of **1**.

**Table 2:** Static first hyperpolarizability *β*(0) tensor components in atomic units (au) for the Zn3-moiety under vacuum, calculated at the B3LYP level with the 6-31 + G(d,p) basis set for C, H, N, and Cl and the SDD basis set in conjunction with the SDD pseudopotential for Zn. Orientation of the molecule is shown in Fig. S12 in the ESI

Tensor components	Computed values (au)	
	Before	After
*β*_xxx_	87 715.4	–7341.62
*β*_xxy_	11 653.8	–282.92
*β*_xyy_	22 153.3	399.19
*β*_yyy_	8986.97	–524.72
*β*_xxz_	–5956.78	399.57
*β*_xyz_	–1430.75	66.66
*β*_yyz_	–1649.61	29.06
*β*_xzz_	2034.63	–509.99
*β*_yzz_	–626.91	–773.48
*β*_zzz_	2662.88	1604.29
Total beta tensor	113 786.41	7884.88

On the other side, the self-absorption effect cannot be ignored. Here, we take the photoswitching of SHG as an example. The SHG-switching contrast after irradiation by the Xe lamp for 60 min was 3 times when the wavelength of the fundamental laser beam was 1800 nm (Fig. 4b). The minor change in absorbance at 900 nm (Fig. 3 left) indicates that the dropping of the SHG intensity was basically contributed by the ET process and the decrease in permanent dipole moment. On the other hand, the SHG-switching contrast after irradiation by the Xe lamp for 60 min reached ∼4 times when the wavelength of the fundamental laser beam was 1064 nm (Fig. 4a). In this case, the change of absorbance at 532 nm was clearly enhanced (Fig. 3 left). Constant irradiation of the irradiated sample for one hour by the 1064 nm laser with an energy of 4 mJ did not show any damage to the NLO crystal (Fig. S13 in the ESI†). That is to say, the increase in self-absorption after coloration facilitates the enhancement of the SHG-switching contrast.

## Conclusions

We have synthesized, through an acentric induced strategy, a new second-order NLO compound showing electron-transfer photochromism and thermochromism in the crystalline state.

It represents the first crystalline compound with both photo- and heat-induced SHG-switching behavior and the first example of a thermoswitchable NLO crystal that can maintain its expected second-order NLO intensity without any heat source. Remarkable decreases in its SHG intensity were observed after photo- or heat-induced coloration. The observed SHG-switching contrast of about 8 times is the highest record for photoswitchable NLO crystals. The decrease in the SHG intensity is contributed by the decrease in the molecular permanent dipole moment, caused by the light/heat-triggered electron-transfer process, and the enhancement of self-absorption. This finding will inspire the design and synthesis of new second-order NLO crystals with higher SHG-switching contrasts through increasing electron-transfer efficiency, variation of molecular permanent dipole moment, and self-absorption.

## Experimental

### Materials and methods

MQCl was synthesized completely according to a reported procedure.^58^ Other chemicals of A.R. grade were obtained from commercial sources and used without further purification. A xenon lamp system equipped (∼112 mW cm^–2^) with an IR filter was used to prepare colored samples for UV-vis absorption, PXRD, ESR, and FT-IR. The Xe lamp and the 355 nm DPSS laser were used for SHG studies. UV-vis spectra were measured in the diffuse reflectance mode using a Perkin-Elmer Lambda 900 UV-vis spectrophotometer equipped with an integrating sphere attachment and BaSO_4_ as a reference. PXRD patterns were collected on a Rigaku DMAX-2500 diffractometer at 40 kV, 100 mA for Cu *K*_α_ radiation at room temperature (*λ* = 1.5406 Å; 2*θ*_max_ = 50°). ESR signals were recorded in the X band at room temperature on a Bruker A300 spectrometer. FT-IR spectra were recorded on a VERTEX 70/70v FT-IR spectrometer over the range of 4000–400 cm^–1^. The photoresponsive range was measured using a 450 W Xe lamp from a single-grating Edinburgh EI920 fluorescence spectrometer. Powder SHG measurements were performed on a modified Kurtz–Perry powder system^59^ using 1064 and 1800 nm fundamental laser beams (pulse width, 10 ns; frequency, 10 Hz). The sample, with a thickness of about 0.2 mm for SHG measurement, was placed in a mould. The SHG signal was collected and focused into a fibre optic bundle, and then coupled to the entrance slit of a spectrometer and detected using a CCD detector. A powdered KDP sample was used as a reference to assume the second-order NLO effect. Both crystalline samples of KDP and compound **1** were ground and sieved into different particle size ranges (30–50, 50–75, 75–100, 100–150, 150–200, and 200–250 μm) for phase-matching measurements according to the literature method.^59^ TG and DSC analyses were done on a NETZSCH STA 449C simultaneous thermal analyzer with Al_2_O_3_ crucibles under N_2_ at a heating rate of 10 °C min^–1^. Elemental analyses of C, H, and N were determined using a Vario EL III CHN elemental analyzer.

### Single-crystal X-ray crystallography

Single-crystal X-ray diffraction data were measured at 293(2) K on a Rigaku Pilatus CCD diffractometer, using graphite monochromated Mo *K*_*α*_ radiation (*λ* = 0.71073 Å). Intensity data sets were collected using a *ω*-scan technique and corrected for *Lp* effects. The structure was solved by a direct method and refined by full-matrix least squares on *F*^2^ using the Siemens SHELXTL™ Version 5 package of crystallographic software,^60^ with anisotropic thermal parameters for all non-hydrogen atoms. Hydrogen atoms were added geometrically and refined using the riding model. The structure was finally verified using the ADDSYM algorithm from the program PLATON.^61^ Crystallographic data and structural refinements for **1** are summarized in Table S6 in the ESI.† The main bond lengths and angles for **1** are shown in Table S7 in the ESI.† More details on the crystallographic studies as well as atomic displacement parameters are given as cif in the ESI.†

The CCDC-1519422 entry contains the supplementary crystallographic data for **1**.†

### Synthesis of β-[(MQ)ZnCl_3_] (**1**)

A mixture of D-Cam (80 mg, 0.4 mmol), Na_2_CO_3_ (42 mg, 0.4 mmol) and H_2_O (6 mL) was added into a 23 mL Teflon-lined autoclave and stirred for 15 min. After this, ZnSO_4_·7H_2_O (144 mg, 0.5 mmol; excess) and MQCl (99 mg, 0.5 mmol) were added into the mixed solution. The vessel was then sealed and heated at 120 °C for 2 d. After cooling to room temperature naturally, the mixed solution was filtered. Pale yellow prismatic crystals of **1** were obtained by slowly evaporating the filtrate for several days, washing with water several times and drying in air. Yield: 60% (based on MQCl). Anal. calcd (%) for C_11_H_11_ZnN_2_Cl_3_: C, 38.49; H, 3.21; N, 8.16. Found: C, 38.69; H, 3.33; N, 8.20.

Without the presence of D-Cam, compound **1** can also be obtained through directly evaporating the filtrate of a mixture of ZnSO_4_·7H_2_O, MQCl and H_2_O with the same molar ratio, but there are only several crystals produced and most of the products are in the centrical phase α-[(MQ)ZnCl_3_].^45^ This means that D-cam is an effective acentric inducer for the synthesis of **1**. The experimental PXRD pattern at room temperature for **1** matched well with the simulated one from the X-ray single-crystal structure data (Fig. S3 in the ESI†). This result and the elemental analysis data of C, H and N demonstrated the phase purity of the obtained crystalline product of **1**.

### Computational details

The refractive indices of **1** were calculated at the GGA/RPBE level using the CASTEP module implemented in the Material Studio software^62^ in a unit cell built directly from its single-crystal X-ray diffraction data. Other calculations were performed using the Gaussian 09 software.^63^ The 6-31 + G(d,p) basis set was used for C, H, N, and Cl, and the SDD basis set in conjunction with the SDD pseudopotential were used for Zn when running the Gaussian calculations. To obtain the values of the permanent dipole moment and Δ*G*, the ground state geometries in the gas phases of MQ^+^ and CEbpy as well as their one-electron reduced species were optimized before frequency calculations with the B3LYP approach. The colored state of **1** after photo/heat-induced electron transfer is a stable ground state instead of an excited state. The product is a charge-separated state which belongs to either a triplet state (*S* = 3) or an open-shell singlet state (*S* = 1). The open-shell singlet state does not exist because no spin density can be found (Table S8 in the ESI†). So, *S* of the initial and final states for **1** were set to 1 and 3, respectively. Optimized structures for the singlet and triplet states of the Zn3-moiety, with the B3LYP, M06, and HF approaches, had large structural distortion compared with the geometry in the crystal (Fig. S14 in the ESI†). However, the closely packed crystal structure did not allow a large structural distortion. In addition, it has been reported that only a minor structural change happens after coloration for viologen-based photochromic compounds.^31^–^33^ So, the geometry of each component in the crystal structure of **1** was used directly for calculations of the permanent dipole moments and first hyperpolarizabilities before and after electron transfer. The B3LYP and M06 approaches were used for the calculations of the permanent dipole moments and first hyperpolarizabilities because of the similarity between the experimental and calculated electron absorption data (Fig. S15 in the ESI†).

## Conflicts of interest

There are no conflicts to declare.

## Supplementary Material

Supplementary information

Crystal structure data
