# Supplementary material for: Second-order nonlinear optical switching with a record-high contrast for a photochromic and thermochromic bistable crystal
Source: Chem Sci. 2017 Sep 20;8(11):7751–7. doi: 10.1039/c7sc01228d (PMC6118235; doi:10.1039/c7sc01228d)
Supplement: Supplementary file 1 [file SC-008-C7SC01228D-s001.pdf]

Electronic Supplementary Material (ESI) for Chemical Science.
This journal is © The Royal Society of Chemistry 2017

Electronic Supplementary Information

Second-order nonlinear optical switch with the record-high contrast for a photochromic and thermochromic bistable crystal

Xiu-Shuang Xing,[a,b] Rong-Jian Sa,[a] Pei-Xin Li,[a] Ning-Ning Zhang,[a,b] Zhong-Yuan Zhou,[a] Bin-Wen Liu,[a] Jie Liu,[a] Ming-Sheng Wang*[a] and Guo-Cong Guo*[a]

[a] *State Key Laboratory of Structural Chemistry, Fujian Institute of Research on the Structure of Matter, Chinese Academy of Sciences, Fuzhou, Fujian 350002, P. R. China.*

[b]*University of Chinese Academy of Sciences, Beijing 100039, P. R. China.*

* *E-mail: gcguo@fjirsm.ac.cn, mswang@fjirsm.ac.cn.*

Table of contents

Table S1 The changes of Gibbs energy (*G*; 1 Hartree = 27.2107 eV) and dipole (*D*) of MQ^+ and CEbpy before and after receiving one electron, respectively.

Table S2. Transition dipole moment, transition energies and oscillator strengths of ground to exited states and exited to exited states for the Zn3-moiety of compound **1** (Eλ = 1240).

Table S3 Static first hyperpolarizability $\beta(0)$ tensor components in atomic unit (au) for the Zn3-moiety in the crystal, calculated at the M06 level with the 6-31+G(d,p) basis set for C, H, N, and Cl and the SDD basis set in conjunction with the SDD pseudopotential for Zn. The molecular structure was not optimized.

Table S4. First SHG [β (-2ω; ω, ω)] hyperpolarizability in atomic unit (au) at λ = 1800 nm fundamental wavelength for the isolated Zn3-moiety in the crystal, calculated at the B3LYP level with the 6-31+G(d,p) basis set for C, H, N, and Cl and the SDD basis set in conjunction with the SDD pseudopotential for Zn. The molecular structure was not optimized.

Table S5. First SHG [β (-2ω; ω, ω)] hyperpolarizability in atomic unit (au) at λ = 1800 nm fundamental wavelength for the isolated Zn3-moiety in the crystal, calculated at the M06 level with the 6-31+G(d,p) basis set for C, H, N, and Cl and the SDD basis set in conjunction with the SDD pseudopotential for Zn. The molecular structure was not optimized.

Table S6 Crystallographic data for **1**.

Table S7 Selected bond lengths of **1**.

Table S8 Spin densities of the Zn3-moiety at the open-shell singlet and triplet diradical states.

Fig. S1 Test of photoresponsive ranges of compound **1**. Each sample was irradiated for 20 min at respective wavelength by a 450 W Xe lamp in a single-grating Edinburgh EI920 fluorescence spectrometer.

Fig. S2 TGA and DSC curves of **1** in N_2.

Fig. S3 PXRD patterns of **1** before irradiation (**1A**), after irradiation (**1P**), and after heat-induced decoloration (**Decolored**). The simulated one is obtained from the single-crystal X-ray diffraction data.

Fig. S4 FT-IR spectra of **1** in the KBr matrix before irradiation (**1A**), after irradiation (**1P**) and after heat-induced decoloration (**Decolored**).

Fig. S5 PXRD patterns of **1** before annealing (**1A**), after annealing at 60 °C (**1T**), and after annealing at 150 °C (**Decolored**). The simulated one is obtained from the single-crystal X-ray diffraction data.

Fig. S6 FT-IR spectra of **1** in the KBr matrix before annealing (**1A**), after annealing at 60 °C (**1T**), and after annealing at 150 °C (**Decolored**).

Fig. S7 Phase matching curve for **1**. Particle sizes are 30–50, 50–75, 75–100, 100–150, 150–200, and 200–250 μm, respectively.

Fig. S8 Calculation of refractive indices n for **1**: **(a)** The direction of the calculated unit cell, where z is along the c axis and y is along the b axis; **(b)** n_x, n_y, and n_z profiles as a function of the energy (eV) of incident light.

Fig. S9 UV–Vis absorption spectra of **1** before irradiation (**1A**), immediately after irradiation (**1P**), and after keeping the irradiated sample in dark under N_2 for 1 d (**1P**-1d).

Fig. S10 UV–Vis spectra of the Zn3-moiety at the closed-shell singlet and open-shell triplet states, calculated by the TD-DFT method using the B3LYP functional with the 6-31+G(d,p) basis set for C, H, N, and Cl and the SDD basis set in conjunction with the SDD pseudopotential for Zn. The molecular structure was not optimized.

Fig. S11 Dipole moments vectors for different components of **1** at the closed-shell singlet and open-shell triplet states. All components were directly truncated from the crystal structure.

Fig. S12 Orientation of the Zn3-moeity for first hyperpolarizability calculations.

Fig. S13 SHG intensities of **1P** after irradiation for 0, 10, 20, 30, 40, 50, 60 min by a fundamental laser at 1064 nm with energy of 4 mJ.

Fig. S14 Molecular structure from the crystal and optimized geometries (B3LYP/6-31+G**, Hartree-Fock/6-31+G**, and M06-2X/6-31+G**) for the Zn3-moiety before and after ET.

Fig. S15 Experimental UV–Vis absorption spectra of **1** and calculated oscillator strengths for the Zn3-moiety that is directly truncated from the crystal structure. The calculations were performed with the TD-DFT method using the M06 or B3LYP functional with the 6-31+G(d,p) basis set for C, H, N, and Cl and the SDD basis set in conjunction with the SDD pseudopotential for Zn.

Fig. S16 Optical absorption spectrum of **1** transformed from the diffuse reflectance data before irradiation.

Full reference for ref. 63 in the manuscript.

Table S1. The changes of Gibbs energy (G; 1 Hartree = 27.2107 eV) and dipole (D) of MQ^+ and CEbpy before and after receiving one electron, respectively.

Compound	G / Hartrees		ΔG /eV	Dipole /Debye		ΔD /Debye
	Before	After		Before	After	
**MQ^+**	–534.934551	–535.133826	**–5.42**	10.36	7.74	**–2.62**
**CEbpy**	–723.105620	–723.188514	**–2.26**	10.10	9.62	**–0.48**

Table S2. Transition dipole moment, transition energies and oscillator strengths of ground to exited states and exited to exited states for the Zn3-moiety of compound **1** (Eλ = 1240).

	states		Transition dipole moment			Diff. (eV)	Oscillator strengths
	i[a]	j[a]	X	Y	Z		
**Before**	0	9	–0.0135	0.0102	–0.0008	1.171	0
	0	10	1.0186	–0.21	0.221	1.2629	0.035
	1	9	–0.0098	0.1023	0.029	1.1288	0.0003
	1	10	–0.1676	–0.0201	1.229	1.2207	0.0460
	2	9	–0.0009	–0.1134	–0.0643	1.0424	0.0004
	2	10	–0.2709	–1.521	0.3775	1.1343	0.0702
	3	9	–0.0419	–0.2762	–0.049	1.0074	0.0020
	3	10	0.4593	–0.2279	–0.8622	1.0993	0.0271
**After**	0	9	–0.0106	–0.0846	–0.0232	1.1978	0.0002
	0	10	–0.0548	–0.0696	0.1042	1.2679	0.0006
	1	9	–0.0029	–0.0045	0.0079	1.1508	0.0000
	1	10	–0.0257	0.076	–0.1584	1.2209	0.0009
	2	9	–0.0021	0.0127	0.0024	1.1016	0.0000
	2	10	–0.087	0.3009	–0.0337	1.1717	0.0028
	3	9	–0.0061	0.0037	–0.0041	0.09874	0.0000
	3	10	0.1785	0.1897	–0.0536	1.0575	0.00183

Table S3 Static first hyperpolarizability $\beta(0)$ tensor components in atomic unit (au) for the Zn3-

moiety in the crystal, calculated at the M06 level with the 6-31+G(d,p) basis set for C, H, N, and Cl and the SDD basis set in conjunction with the SDD pseudopotential for Zn. The molecular structure was not optimized.

Tensor Components	Computed values (au)	
	Before	After
β_{xxx}	344971	–9306.19
β_{xxy}	–41242.7	0.56
β_{xyy}	23104.1	513.09
β_{xyy}	–460.17	–41.86
β_{yyy}	–14178.7	327.23
β_{xxz}	2447.01	–45.28
β_{yyz}	–945.6	–610.1
β_{xzz}	3277.1	–62.36
β_{yzz}	236.62	1.72
β_{zzz}	2636.7	110.08
**Total beta tensor**	**373868.76**	**8857.24**

Table S4. First SHG [β (–2ω; ω, ω)] hyperpolarizability in atomic unit (au) at λ = 1800 nm fundamental wavelength for the isolated Zn3-moiety in the crystal, calculated at the B3LYP level with the 6-31+G(d,p) basis set for C, H, N, and Cl and the SDD basis set in conjunction with the SDD pseudopotential for Zn. The molecular structure was not optimized.

Tensor Components	Computed values (au)	
	Before	After
β_{xxx}	–752723	–14479.2
β_{yxx}	25180	–116655
β_{zxx}	–241715	15291.6
$\beta_{xyx} = \beta_{xxy}$	–8383.5	4235.26
$\beta_{yyx} = \beta_{yxy}$	–7167.3	–71519.7
$\beta_{zyx} = \beta_{zxy}$	–8378.01	–3874.53
β_{xyy}	–17098.8	5961.39
β_{yyy}	–3468.76	16364.7
β_{zyy}	–9888.1	–12219.7
$\beta_{xzx} = \beta_{xxz}$	–48373.4	3790.79
$\beta_{yzx} = \beta_{yxz}$	2047.11	96837.5

$\beta_{zzx} = \beta_{zxz}$	–10298.9	–11597.7
βxzy=βxyz	–4445.98	–4075.37
$\beta_{yzy} = \beta_{yyz}$	–1606.09	–3217.06
$\beta_{zzy} = \beta_{zyz}$	–2816.13	7882.09
β_{xzz}	1495.69	–818.55
β_{yzz}	1265.89	19968.4
β_{zzz}	2836.07	754.5
**Total beta tensor**	**778020.49**	**68653.68**

Table S5. First SHG [β (–2ω; ω, ω)] hyperpolarizability in atomic unit (au) at λ = 1800 nm fundamental wavelength for the isolated Zn3-moiety in the crystal, calculated at the M06 level with the 6-31+G(d,p) basis set for C, H, N, and Cl and the SDD basis set in conjunction with the SDD pseudopotential for Zn. The molecular structure was not optimized.

Tensor Components	Computed values (au)	
	Before	After
β_{xxx}	–294930	10904.3
β_{yxx}	–25811.1	–55882.3
β_{zxx}	–143942	–38316.5
$\beta_{xyx} = \beta_{xxy}$	–14289	6679.01
$\beta_{yyx} = \beta_{yxy}$	–19055.1	–13325.5
$\beta_{zyx} = \beta_{zxy}$	–23875.2	–9723.24
β_{xyy}	–13083.6	58873.3
β_{yyy}	–7538.51	–61192.3
β_{zyy}	–11776.4	–106871
$\beta_{xzx} = \beta_{xxz}$	63631.3	7713.75
$\beta_{yzx} = \beta_{yxz}$	6519.87	3375.39
$\beta_{zzx} = \beta_{zxz}$	29507	–15881.5
β_{xzy}=β_{xyz}	3231.28	–32183.9
$\beta_{yzy} = \beta_{yyz}$	–4490.34	35880.4
$\beta_{zzy} = \beta_{zyz}$	–3249.91	57622.3
β_{xzz}	48315.2	–18868
β_{yzz}	4526.22	30420.6
β_{zzz}	21564.5	34548.6
**Total beta tensor**	**277618.63**	**31195.03**

Table S6. Crystallographic data for **1**.

Empirical formula	$ZnC_{11}H_{11}Cl_3N_2$
**Formula weight**	342.94
**Temperature (K)**	293(2)
**Crystal system**	Monoclinic
**Space group**	*Cc*
**Flack factor**	–0.02(2)
a (Å)	13.791(3)
b (Å)	21.838(4)
c (Å)	14.457(3)
β (°)	107.005(5)
V (Å^3)	4163.6(15)
Z	12
**Crystal size (mm^3)**	0.41 × 0.35 × 0.25
F(000)	2064
θ **range for data collection (°)**	2.599–27.476
D_{calcd} (g cm^{-3})	1.641
μ (mm^{-1})	2.33
**GOF on** F^2	0.98
$R_1{}^a$ $[I > 2\sigma(I)]$	0.039
$wR_2{}^b$ $[I > 2\sigma(I)]$	0.141
$\Delta\rho_{max}/\Delta\rho_{min}$(e Å^{-3})	0.40/–0.80

[a] $R_1 = \sum||F_o|–|F_c||/\sum|F_o|$; [b] $wR_2 = \sum[w(F_o{}^2–F_c{}^2)^2]/\sum[w(F_o{}^2)^2]^{1/2}$

Table S7. Selected bond lengths of **1**.

Label	Distances (Å)	Label	Distances (Å)
**Zn1—N12**	2.073 (3)	**C29—C210**	1.388 (6)
**Zn1—Cl13**	2.2245 (14)	**N21—C23**	1.337 (6)
**Zn1—Cl12**	2.2433 (14)	**N21—C22**	1.331 (6)
**Zn1—Cl11**	2.2554 (15)	**N21—C211**	1.480 (6)
**N11—C12**	1.323 (6)	**N22—C29**	1.327 (6)
**N11—C13**	1.339 (6)	**N22—C28**	1.338 (5)
**N11—C111**	1.500 (6)	**C21—C22**	1.367 (6)

**C11—C12**	1.367 (7)	**C21—C25**	1.389 (6)
**C11—C15**	1.379 (6)	**C23—C24**	1.361 (6)
**N12—C19**	1.345 (6)	**Zn3—N32**	2.080 (3)
**N12—C18**	1.333 (5)	**Zn3—Cl33**	2.2298 (16)
**C13—C14**	1.365 (6)	**Zn3—Cl32**	2.2277 (15)
**C14—C15**	1.391 (6)	**Zn3—Cl31**	2.2608 (13)
**C15—C16**	1.482 (6)	**N31—C33**	1.333 (7)
**C16—C110**	1.382 (6)	**N31—C32**	1.335 (6)
**C16—C17**	1.399 (6)	**N31—C311**	1.476 (7)
**C17—C18**	1.374 (6)	**C31—C32**	1.361 (7)
**C19—C110**	1.375 (6)	**C31—C35**	1.388 (6)
**Zn2—N22**	2.076 (4)	**N32—C38**	1.335 (6)
**Zn2—Cl23**	2.2090 (15)	**N32—C39**	1.337 (6)
**Zn2—Cl21**	2.2261 (14)	**C33—C34**	1.368 (7)
**Zn2—Cl22**	2.2619 (15)	**C34—C35**	1.384 (6)
**C24—C25**	1.376 (6)	**C35—C36**	1.492 (6)
**C25—C26**	1.482 (5)	**C36—C310**	1.388 (6)
**C26—C27**	1.385 (6)	**C36—C37**	1.381 (6)
**C26—C210**	1.383 (6)	**C37—C38**	1.380 (6)
**C27—C28**	1.372 (6)	**C39—C310**	1.369 (6)

Table S8. Spin densities of the Zn3-moiety at the open-shell singlet and triplet diradical states.

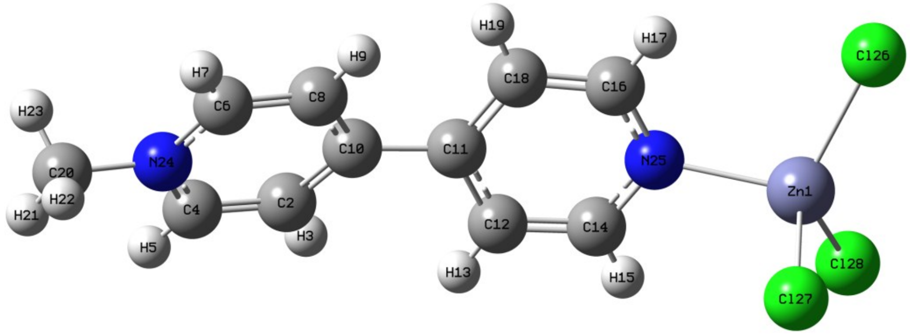

No.	Elements	Spin density	
		Open-shell singlet state	Triplet state
**1**	**Zn**	0	–0.067590
**2**	**C**	0	0.005913
**3**	**H**	0	0.000876
**4**	**C**	0	0.185271
**5**	**H**	0	–0.006881
**6**	**C**	0	0.178472
**7**	**H**	0	–0.006387
**8**	**C**	0	–0.029245
**9**	**H**	0	0.001153
**10**	**C**	0	0.274125
**11**	**C**	0	–0.044953
**12**	**C**	0	0.080234
**13**	**H**	0	–0.002736
**14**	**C**	0	–0.006800
**15**	**H**	0	–0.000061
**16**	**C**	0	–0.010323
**17**	**H**	0	–0.000291
**18**	**C**	0	0.127825
**19**	**H**	0	–0.003054

**20**	**C**	0	–0.009970
**21**	**H**	0	0.000261
**22**	**H**	0	0.008138
**23**	**H**	0	0.007394
**24**	**N**	0	0.160791
**25**	**N**	0	0.095216
**26**	**Cl**	0	0.277955
**27**	**Cl**	0	0.434034
**28**	**Cl**	0	0.350633

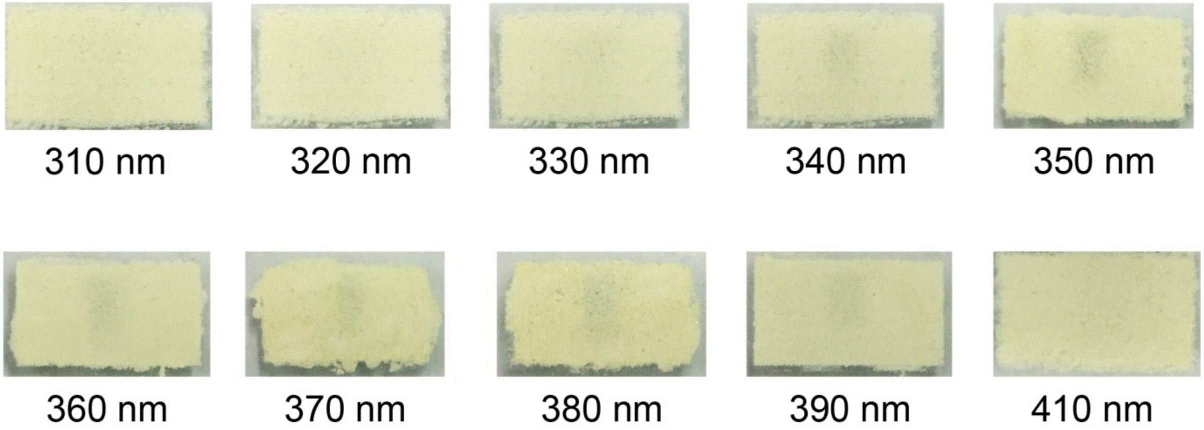

Fig. S1 Test of photoresponsive ranges of compound **1**. Each sample was irradiated for 20 min at respective wavelength by a 450 W Xe lamp in a single-grating Edinburgh EI920 fluorescence spectrometer.

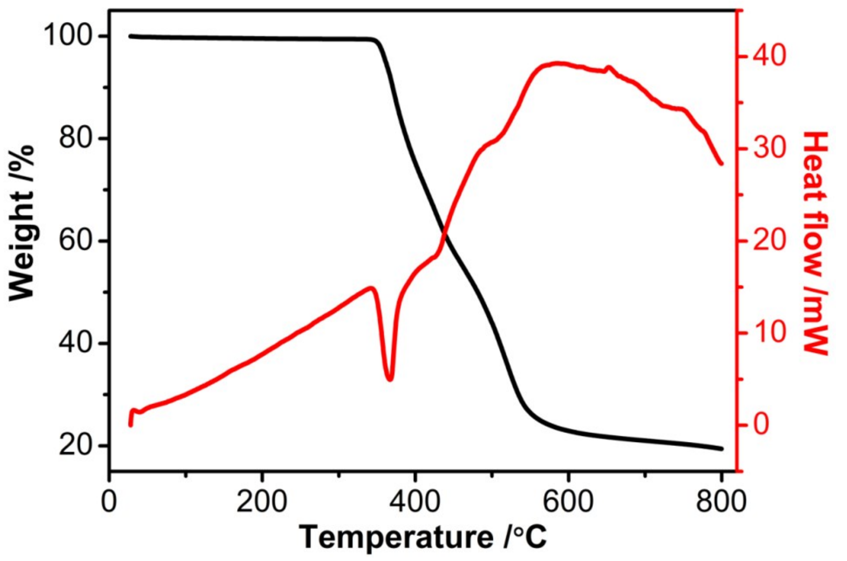

Fig. S2 TGA and DSC curves of **1** in N_2.

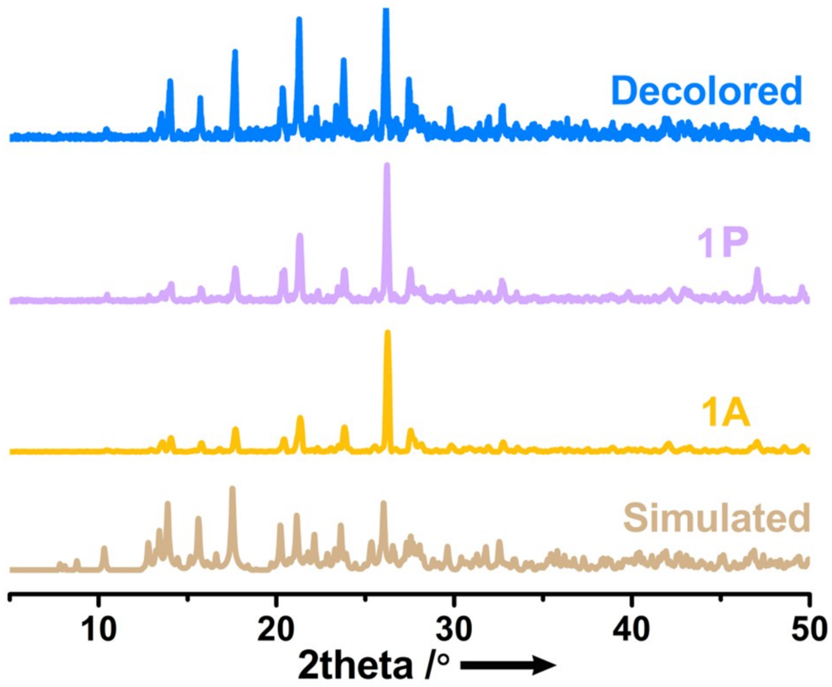

Fig. S3 PXRD patterns of **1** before irradiation (**1A**), after irradiation (**1P**), and after heat-induced decoloration (**Decolored**). The simulated one is obtained from the single-crystal X-ray diffraction data.

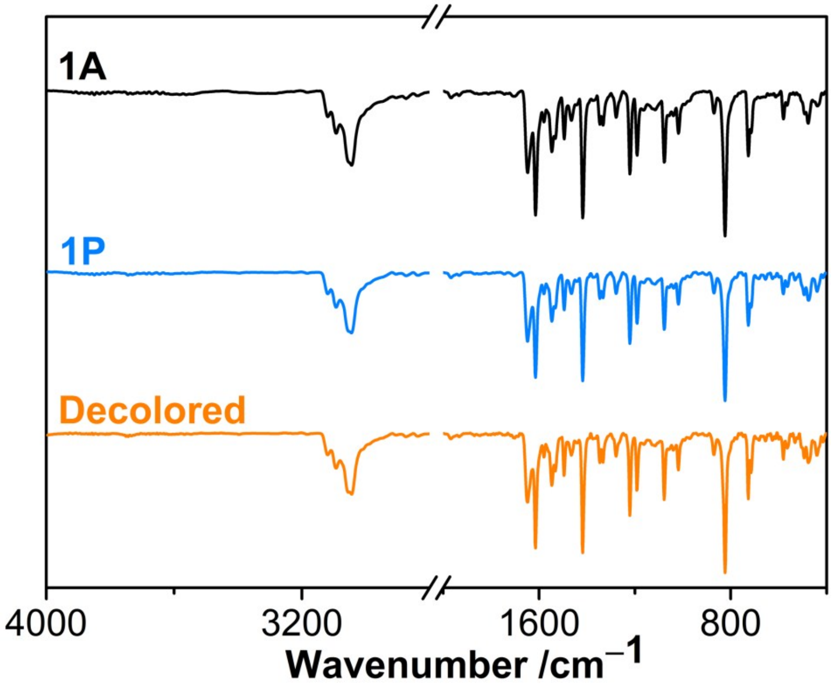

Fig. S4 FT-IR spectra of **1** in the KBr matrix before irradiation (**1A**), after irradiation (**1P**) and after heat-induced decoloration (**Decolored**).

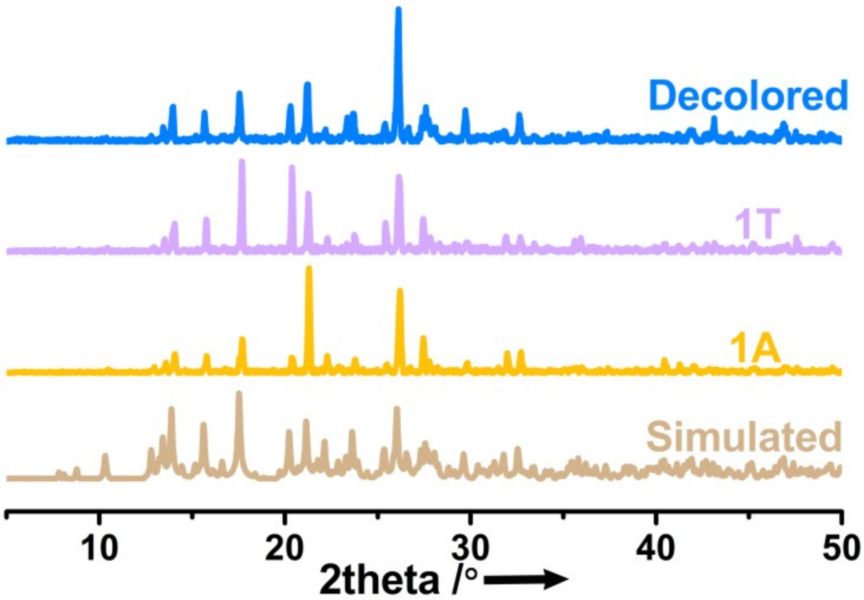

Fig. S5 PXRD patterns of **1** before annealing (**1A**), after annealing at 60 °C (**1T**), and after annealing at 150 °C (**Decolored**). The simulated one is obtained from the single-crystal X-ray diffraction data.

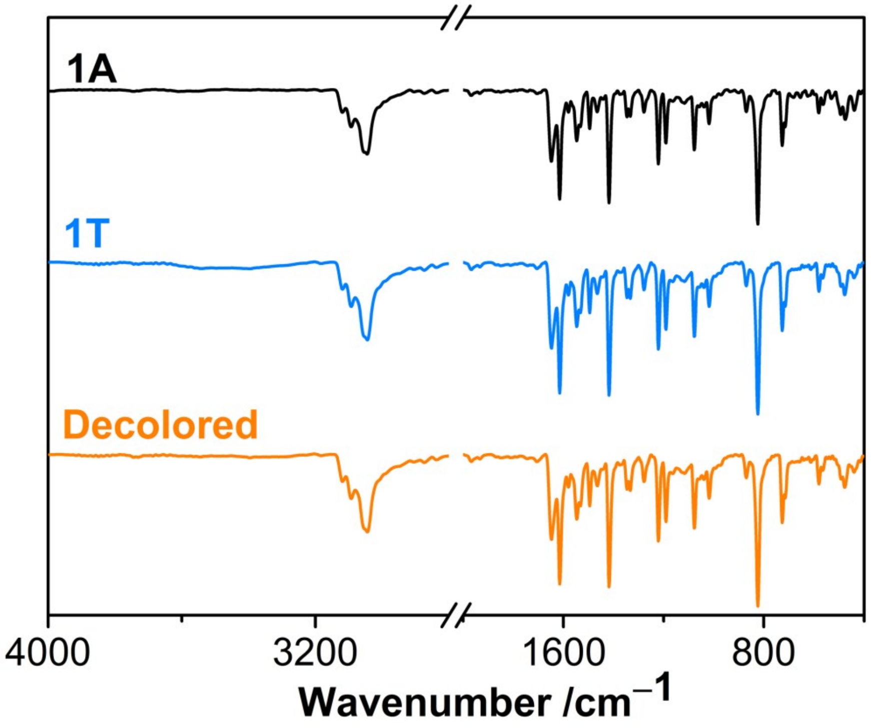

Fig. S6 FT-IR spectra of **1** in the KBr matrix before annealing (**1A**), after annealing at 60 °C (**1T**), and after annealing at 150 °C (**Decolored**).

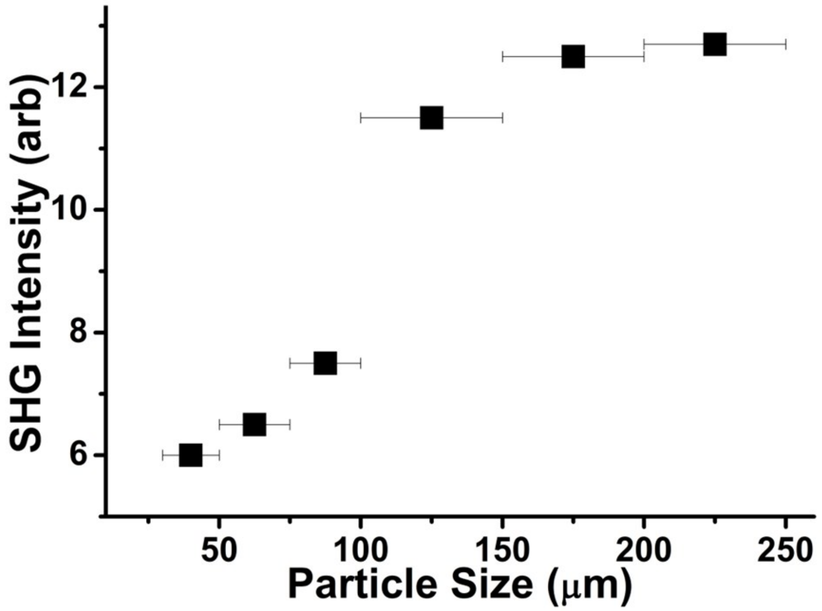

Fig. S7 Phase matching curve for **1**. Particle sizes are 30–50, 50–75, 75–100, 100–150, 150–200, and 200–250 μm, respectively.

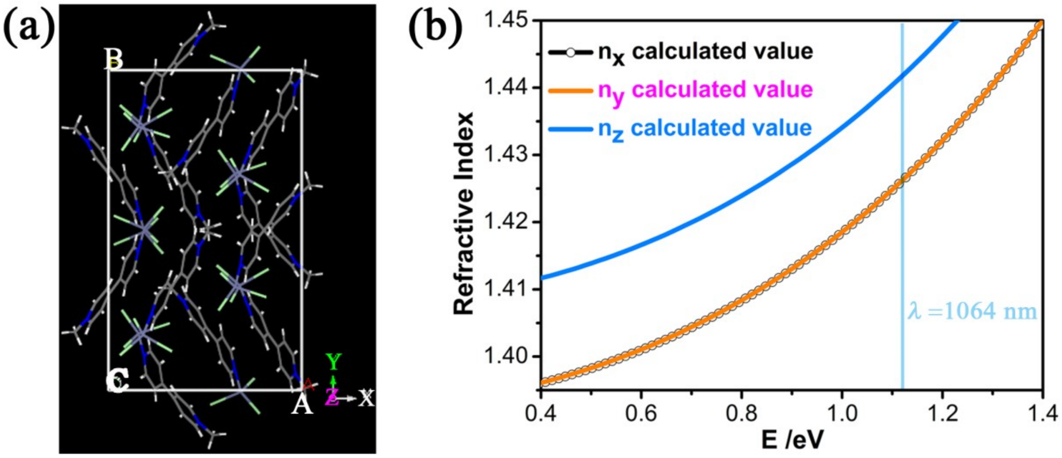

Fig. S8 Calculation of refractive indices *n* for **1**: **(a)** The direction of the calculated unit cell, where z is along the c axis and y is along the b axis; **(b)** n_x, n_y, and n_z profiles as a function of the energy (eV) of incident light.

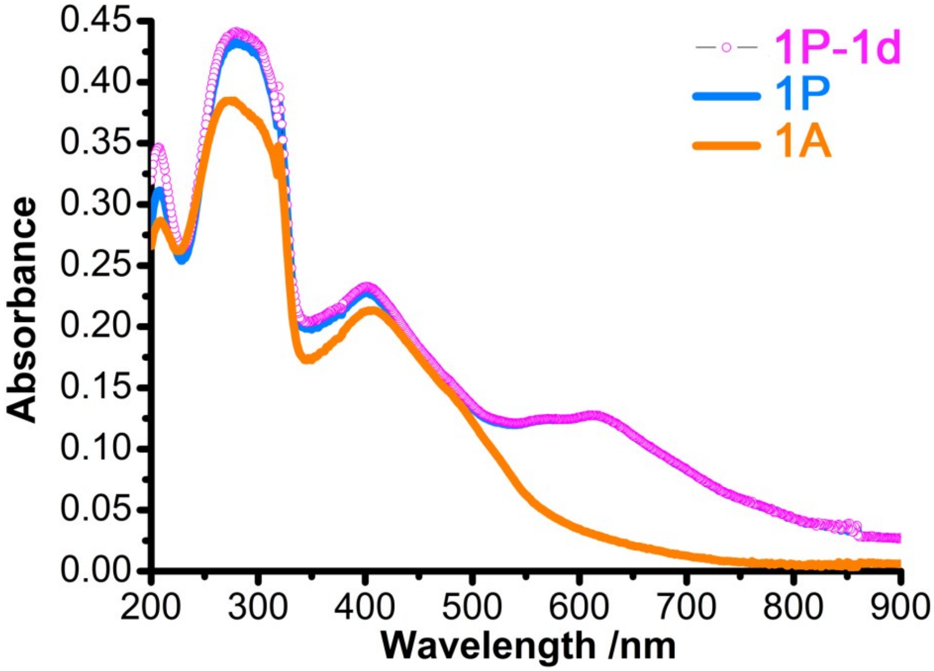

Fig. S9 UV–Vis absorption spectra of **1** before irradiation (**1A**), immediately after irradiation (**1P**), and after keeping the irradiated sample in dark under N_2 for 1 d (**1P**-1d).

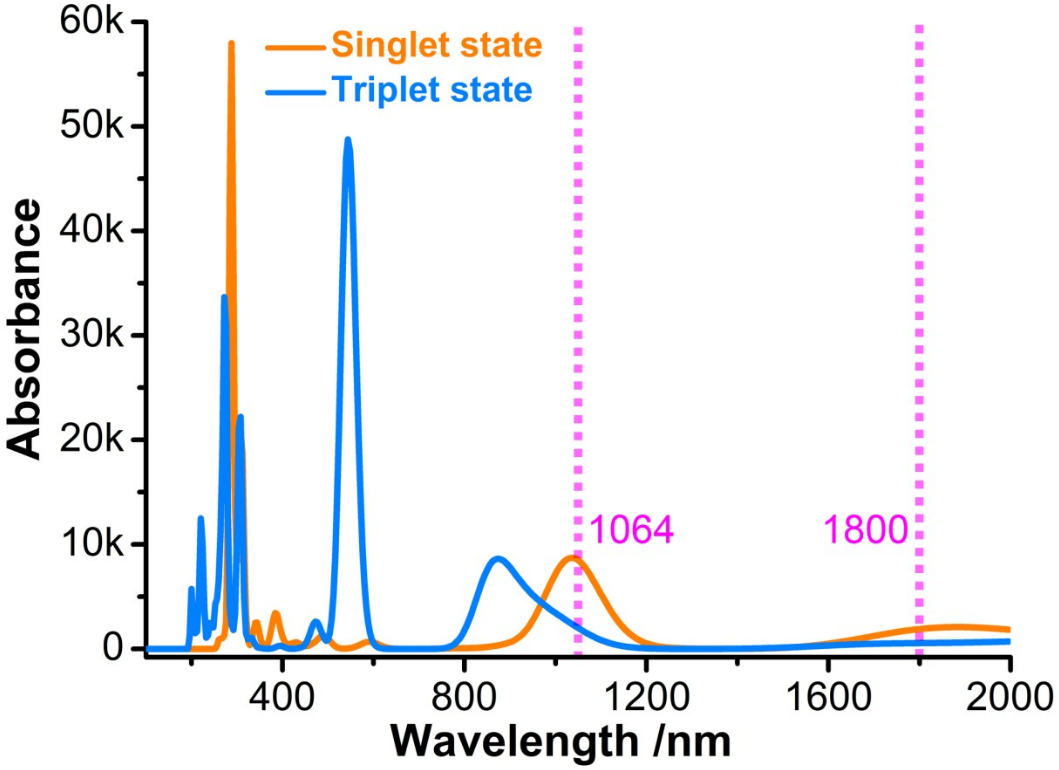

Fig. S10 UV–Vis spectra of the Zn3-moiety at the closed-shell singlet and open-shell triplet states, calculated by the TD-DFT method using the B3LYP functional with the 6-31+G(d,p) basis set for C, H, N, and Cl and the SDD basis set in conjunction with the SDD pseudopotential for Zn. The molecular structure was not optimized.

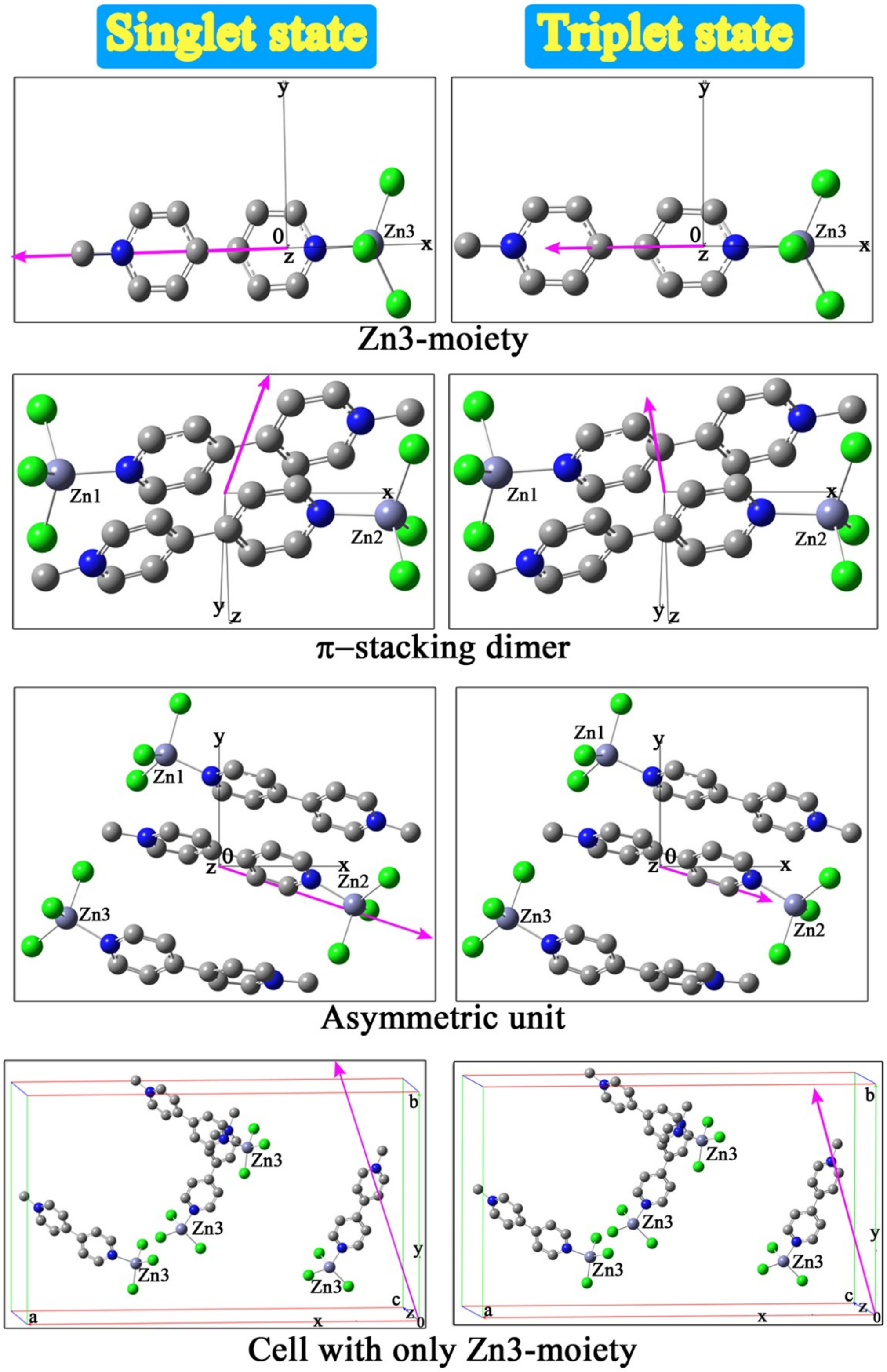

Fig. S11 Dipole moments vectors for different components of **1** at the closed-shell singlet and open-shell triplet states. All components were directly truncated from the crystal structure.

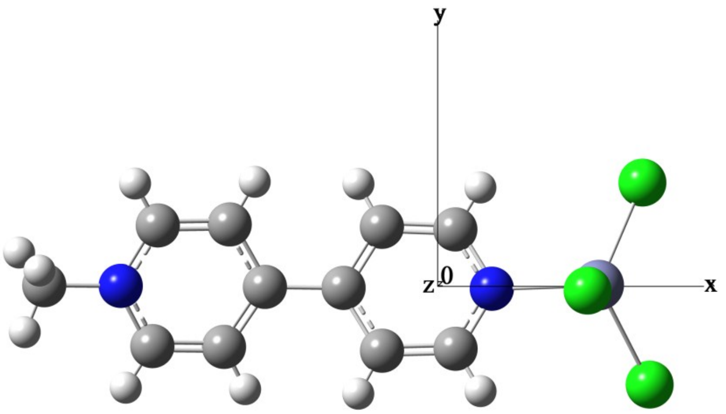

Fig. S12 Orientation of the Zn3-moeity for first hyperpolarizability calculations.

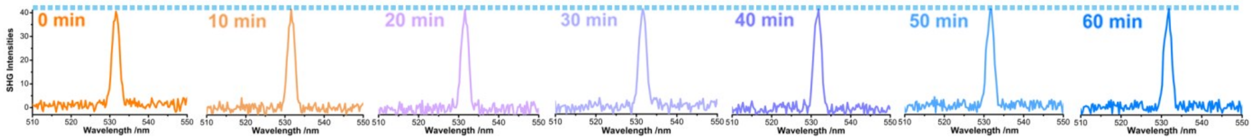

Fig. S13 SHG intensities of **1P** after irradiation for 0, 10, 20, 30, 40, 50, 60 min by a fundamental laser at 1064 nm with energy of 4 mJ.

(a) From the crystal

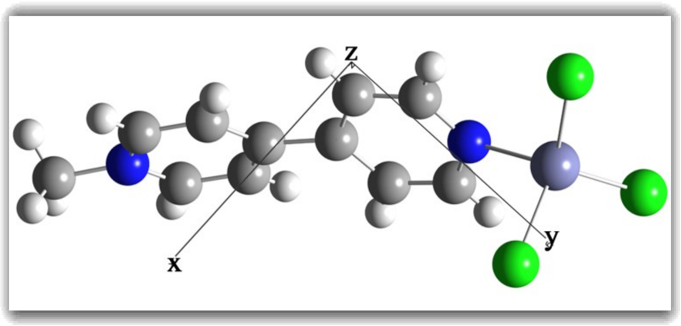

(b) From B3LYP calculation

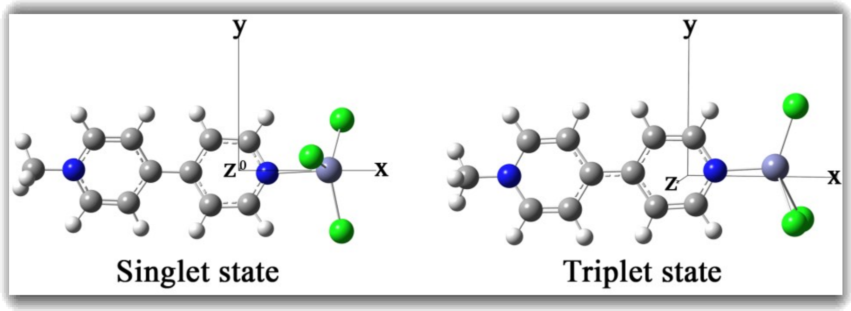

Z-matrix for the singlet state:

Zn	-1.89168000	1.13853000	-0.29803400
C	0.70471800	-0.90720200	1.45916800
H	1.13831500	-0.43096800	2.33328900
C	-0.56334400	-1.43584700	1.54841700
H	-1.16023600	-1.38437900	2.45228000
C	-0.48961400	-2.09538500	-0.70426900
H	-1.03989800	-2.53682000	-1.52869800
C	0.78411200	-1.59288800	-0.83741700
H	1.26197900	-1.62517300	-1.81175000
C	1.41148900	-0.97446800	0.25224300
C	2.76910000	-0.42212200	0.13395400
C	3.12133400	0.77252400	0.76551400
H	2.38088100	1.36099500	1.30273200
C	4.42578100	1.23541800	0.62496500
H	4.72222200	2.17334200	1.09300300
C	5.02518800	-0.54180200	-0.67065400
H	5.80973600	-1.04993700	-1.23087600
C	3.74760900	-1.08818400	-0.60763500
H	3.53219700	-2.03256800	-1.10363600
C	-2.51775700	-2.54540200	0.56207700
H	-2.95815400	-2.22539300	1.50673400
H	-3.09136400	-2.10447300	-0.26069900
H	-2.50058500	-3.63603500	0.48843100
N	-1.14110600	-2.02960300	0.47988800
N	5.37018700	0.59744100	-0.06962600
Cl	0.11141600	2.12778800	-0.40209500
Cl	-2.61595200	-0.13370000	-2.02627500
Cl	-2.87546000	0.76666500	1.70308800

Z-matrix for the triplet state:

Zn	3.03546100	0.31033700	0.00426700
C	-4.06949200	-1.22633000	-0.01169900
H	-3.55577700	-2.17938400	-0.00671500
C	-5.43326200	-1.26027400	-0.01601000
H	-5.99051900	-2.18947500	-0.01158900
C	-5.52751200	1.11046500	-0.02786200
H	-6.15674200	1.99248000	-0.03340100
C	-4.16537300	1.18491500	-0.02330200
H	-3.72876900	2.17575900	-0.02916700
C	-3.33849100	0.01029400	-0.01568200
C	-1.89932500	0.06759900	-0.01119100
C	-1.08412600	-1.10091000	-0.01020900
H	-1.51759800	-2.09345800	-0.01357800
C	0.29210900	-1.00432900	-0.00583800
H	0.90999300	-1.89700800	-0.00535000

C	0.20188400	1.30824500	-0.00288000
H	0.75442100	2.24362500	0.00048100
C	-1.17825000	1.29613900	-0.00733400
H	-1.68823100	2.25160100	-0.00681600
C	-7.64357400	-0.16046300	0.08183000
H	-8.01503000	-1.06795200	-0.39933900
H	-7.95700300	-0.16089400	1.13356300
H	-8.08709800	0.70229700	-0.42025500
N	-6.19059500	-0.10330900	-0.03976800
N	0.95376300	0.17921500	-0.00205000
Cl	3.72348600	2.43888700	0.01239600
Cl	3.83131700	-1.54605900	1.38293600
Cl	3.83924400	-1.53819000	-1.38015800

(c) From Hartree–Fock calculation

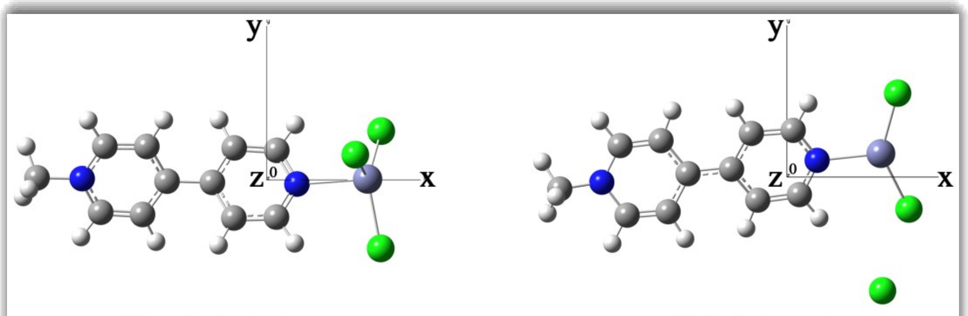

Singlet state

Triplet state

Z-matrix for the singlet state:

Zn	3.27963300	0.02676100	0.09560700
C	-4.03182000	-1.12305200	-0.53946100
H	-3.59235000	-2.00637400	-0.95965300
C	-5.39198700	-1.04973000	-0.41763100
H	-6.03310400	-1.85600100	-0.71664500
C	-5.25752300	1.09233600	0.46536100
H	-5.79407800	1.93162300	0.86331000
C	-3.89375400	1.08526800	0.36797400
H	-3.34099600	1.93790300	0.71075500
C	-3.23740600	-0.04049400	-0.14399900
C	-1.76338800	-0.08196200	-0.25087600
C	-1.06577100	-1.23612500	0.08088600
H	-1.56273700	-2.12308800	0.42767800
C	0.31857300	-1.20699100	0.01725300
H	0.91244700	-2.06081700	0.28682200
C	0.32844900	0.95925800	-0.71133900
H	0.92937600	1.79179300	-1.02772200
C	-1.05371500	1.03919000	-0.66214300

H	-1.54451800	1.94819500	-0.95671000
C	-7.45928500	0.06794600	0.23974000
H	-7.90870300	-0.55501600	-0.51769200
H	-7.72012000	-0.29816200	1.22321100
H	-7.80870800	1.08127400	0.11726700
N	-5.99355400	0.04166900	0.07580300
N	0.98362000	-0.13377200	-0.36667400
Cl	3.72047000	1.58390400	-1.54514700
Cl	2.87957800	0.81776500	2.23412600
Cl	3.70523400	-2.22421700	-0.15705700

Z-matrix for the triplet state:

Zn	3.09689600	0.77362000	-0.18663600
C	-3.91177100	-1.14276700	-0.33622900
H	-3.35128700	-2.03587400	-0.52943900
C	-5.26289900	-1.25518000	-0.31591700
H	-5.75120700	-2.19440700	-0.49107200
C	-5.49338700	1.03620200	0.21055100
H	-6.15831600	1.85206600	0.41931600
C	-4.14737100	1.19985900	0.20864600
H	-3.77631100	2.18444800	0.41348400
C	-3.25652400	0.11087200	-0.08361800
C	-1.82193200	0.25967300	-0.10676900
C	-0.95943400	-0.76142400	-0.57428300
H	-1.33930200	-1.69019800	-0.94932600
C	0.41205500	-0.57915800	-0.57940800
H	1.06877700	-1.34954400	-0.93662800
C	0.20788600	1.53826100	0.29635800
H	0.70600200	2.42711600	0.63500300
C	-1.17140000	1.43762900	0.33450600
H	-1.72056000	2.27148700	0.72271700
C	-7.48291000	-0.39276600	0.25848300
H	-7.88655900	-1.19807500	-0.34155800
H	-7.59892600	-0.64663900	1.31049100
H	-8.05702500	0.50079500	0.04986300
N	-6.09796300	-0.17134800	-0.09864200
N	0.99594200	0.54996100	-0.15232100
Cl	3.64948900	2.76288000	0.61045700
Cl	3.15199700	-3.74585100	1.08136600
Cl	4.02193200	-1.05745700	-1.03423200

(d) From M06 calculation

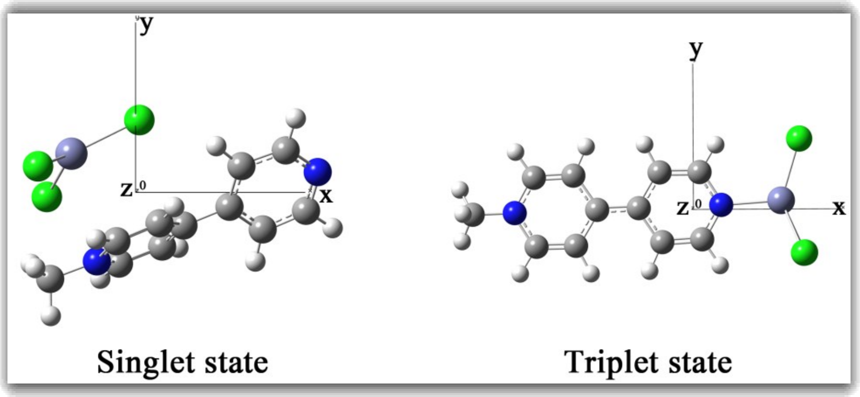

Z-matrix for the singlet state:

Zn	-1.89168000	1.13853000	-0.29803400
C	0.70471800	-0.90720200	1.45916800
H	1.13831500	-0.43096800	2.33328900
C	-0.56334400	-1.43584700	1.54841700
H	-1.16023600	-1.38437900	2.45228000
C	-0.48961400	-2.09538500	-0.70426900
H	-1.03989800	-2.53682000	-1.52869800
C	0.78411200	-1.59288800	-0.83741700
H	1.26197900	-1.62517300	-1.81175000
C	1.41148900	-0.97446800	0.25224300
C	2.76910000	-0.42212200	0.13395400
C	3.12133400	0.77252400	0.76551400
H	2.38088100	1.36099500	1.30273200
C	4.42578100	1.23541800	0.62496500
H	4.72222200	2.17334200	1.09300300
C	5.02518800	-0.54180200	-0.67065400
H	5.80973600	-1.04993700	-1.23087600
C	3.74760900	-1.08818400	-0.60763500
H	3.53219700	-2.03256800	-1.10363600
C	-2.51775700	-2.54540200	0.56207700
H	-2.95815400	-2.22539300	1.50673400
H	-3.09136400	-2.10447300	-0.26069900
H	-2.50058500	-3.63603500	0.48843100
N	-1.14110600	-2.02960300	0.47988800
N	5.37018700	0.59744100	-0.06962600
Cl	0.11141600	2.12778800	-0.40209500
Cl	-2.61595200	-0.13370000	-2.02627500
Cl	-2.87546000	0.76666500	1.70308800

Z-matrix for the triplet state:

Zn	3.01970500	0.29893300	0.00502200
C	-4.04373300	-1.22825700	-0.00670800
H	-3.53142900	-2.18505900	0.00322700
C	-5.40246600	-1.25353900	-0.01019700

H	-5.96927000	-2.18007000	-0.00072000
C	-5.48354600	1.11180300	-0.03317800
H	-6.11263800	1.99718000	-0.04273100
C	-4.12630000	1.17859100	-0.02924800
H	-3.68223100	2.16887700	-0.04149900
C	-3.31183900	0.00216000	-0.01584900
C	-1.87837900	0.05359100	-0.01060900
C	-1.07588200	-1.11520600	-0.01145000
H	-1.51658600	-2.10727800	-0.01815700
C	0.29644000	-1.02473200	-0.00628700
H	0.91044700	-1.92429400	-0.00748200
C	0.21674200	1.28024300	0.00108700
H	0.77798600	2.21498600	0.00658600
C	-1.15891500	1.27552600	-0.00389200
H	-1.66910100	2.23377900	-0.00046700
C	-7.59380900	-0.14429200	0.07980800
H	-7.96895300	-1.06059500	-0.38475900
H	-7.91379200	-0.12327500	1.12997000
H	-8.03569200	0.70954900	-0.44182900
N	-6.14972000	-0.09559800	-0.03737000
N	0.95867100	0.15179200	-0.00005400
Cl	3.65046600	2.41414400	0.01273900
Cl	3.81564500	-1.50300800	1.37834300
Cl	3.81786500	-1.49469300	-1.38000700

Fig. S14 Molecular structure from the crystal and optimized geometries (B3LYP/6-31+G**, Hartree-Fock/6-31+G**, and M06/6-31+G**) for the Zn3-moiety before and after ET.

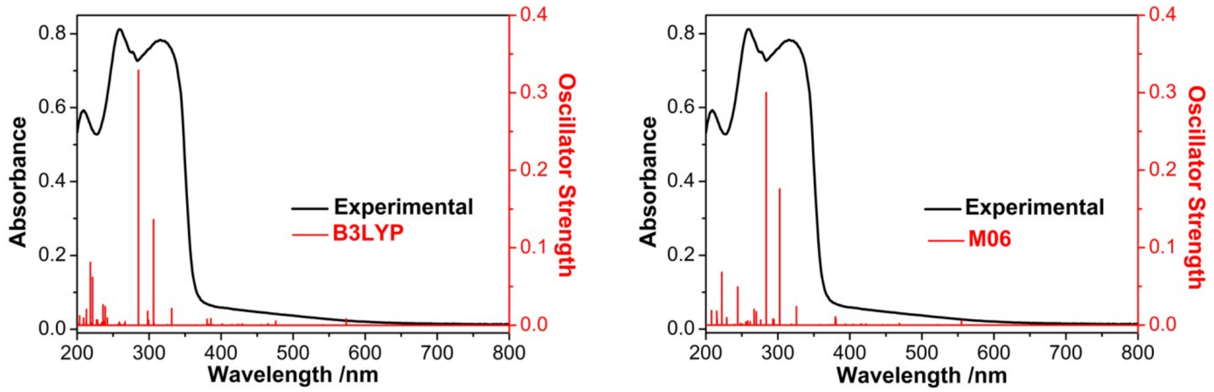

Fig. S15 Experimental UV–Vis absorption spectra of **1** and calculated oscillator strengths for the Zn3-moiety that is directly truncated from the crystal structure. The calculations were performed with the TD-DFT method using the M06 or B3LYP functional with the 6-31+G(d,p) basis set for C, H, N, and Cl and the SDD basis set in conjunction with the SDD pseudopotential for Zn.

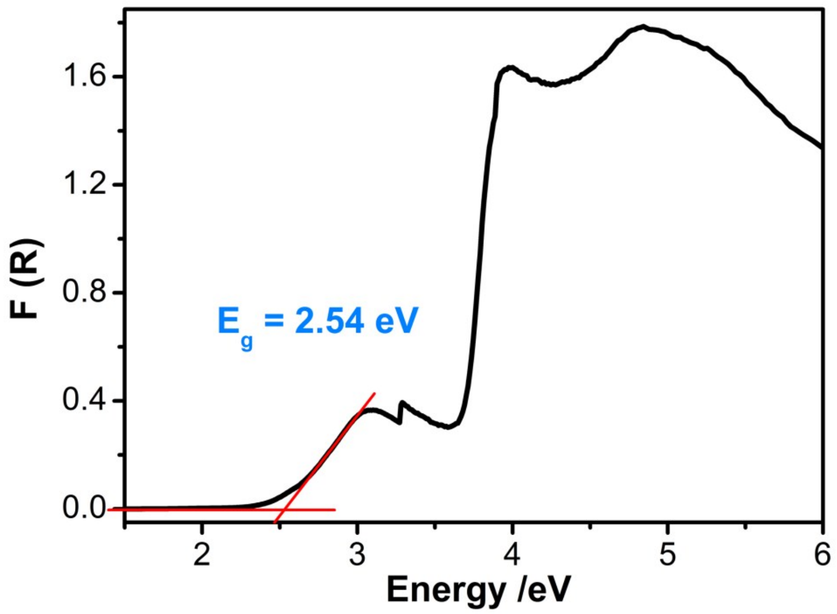

Fig. S16 Optical absorption spectrum of **1** transformed from the diffuse reflectance data before irradiation.

Full reference for ref. 63 in the manuscript:

Gaussian 09, Revision D.01, M. J. Frisch, G. W. Trucks, H. B. Schlegel, G. E. Scuseria, M. A. Robb, J. R. Cheeseman, G. Scalmani, V. Barone, B. Mennucci, G. A. Petersson, H. Nakatsuji, M. Caricato, X. Li, H. P. Hratchian, A. F. Izmaylov, J. Bloino, G. Zheng, J. L. Sonnenberg, M. Hada, M. Ehara, K. Toyota, R. Fukuda, J. Hasegawa, M. Ishida, T. Nakajima, Y. Honda, O. Kitao, H. Nakai, T. Vreven, J. A. Montgomery, Jr., J. E. Peralta, F. Ogliaro, M. Bearpark, J. J. Heyd, E. Brothers, K. N. Kudin, V. N. Staroverov, T. Keith, R. Kobayashi, J. Normand, K. Raghavachari, A. Rendell, J. C. Burant, S. S. Iyengar, J. Tomasi, M. Cossi, N. Rega, J. M. Millam, M. Klene, J. E. Knox, J. B. Cross, V. Bakken, C. Adamo, J. Jaramillo, R. Gomperts, R. E. Stratmann, O. Yazyev, A. J. Austin, R. Cammi, C. Pomelli, J. W. Ochterski, R. L. Martin, K. Morokuma, V. G. Zakrzewski, G. A. Voth, P. Salvador, J. J. Dannenberg, S. Dapprich, A. D. Daniels, O. Farkas, J. B. Foresman, J. V. Ortiz, J. Cioslowski, and D. J. Fox, Gaussian, Inc., Wallingford CT, 2013.
